# A social robot-based platform for health behavior change toward prevention of childhood obesity

**DOI:** 10.1007/s10209-022-00922-7

**Published:** 2022-10-01

**Authors:** Andreas Triantafyllidis, Anastasios Alexiadis, Dimosthenis Elmas, Georgios Gerovasilis, Konstantinos Votis, Dimitrios Tzovaras

**Affiliations:** Information Technologies Institute, Centre for Research and Technology Hellas (CERTH), 57001 Thessaloníki, Greece

**Keywords:** Social robots, Child healthcare, Childhood obesity, Health behavior change, Child–robot interaction

## Abstract

Childhood obesity is a major public health challenge which is linked with the occurrence of diseases such as diabetes and cancer. The COVID-19 pandemic has forced changes to the lifestyle behaviors of children, thereby making the risk of developing obesity even greater. Novel preventive tools and approaches are required to fight childhood obesity. We present a social robot-based platform which utilizes an interactive motivational strategy in communication with children, collects self-reports through the touch of tangible objects, and processes behavioral data, aiming to: (a) screen and assess the behaviors of children in the dimensions of physical activity, diet, and education, and (b) recommend individualized goals for health behavior change. The platform was integrated through a microservice architecture within a multi-component system targeting childhood obesity prevention. The platform was evaluated in an experimental study with 30 children aged 9–12 years in a real-life school setting, showing children’s acceptance to use it, and an 80% success rate in achieving weekly personal health goals recommended by the social robot-based platform. The results provide preliminary evidence on the implementation feasibility and potential of the social robot-based platform toward the betterment of children’s health behaviors in the context of childhood obesity prevention. Further rigorous longer-term studies are required.

## Introduction

Childhood obesity is associated with the prevalence of obesity in adult life and the occurrence of many chronic conditions, including cardiovascular disease, diabetes and cancer [1]. According to the World Health Organization (WHO) [2], it is estimated that 1 in 3 children at the age of 11 in Europe, is overweight or obese. Furthermore, the recent COVID-19 pandemic had an enormous impact on the lifestyle behaviors of children [3, 4], thereby increasing the risk of developing obesity. In this context, preventing the onset of childhood obesity emerges as a crucial challenge. Novel strategies and approaches are required which can promote the fight against childhood obesity through the adoption of healthy behaviors, such as a balanced diet [5] and regular physical activity [6].

Social or socially interactive robots are robots for which social human–robot interaction is important [7]. Social robots have emerged as useful agents in promotion of healthy behaviors for children, due to their engaging characteristics and natural interaction capabilities, which may promote persuasion [8, 9]. Although various web sites, mobile apps and virtual agents have been developed in this scope, their potential to encourage adherence to healthy behaviors has been questioned [10], mainly because of their limited interactivity [11]. On the contrary, social robot-based systems can provide engagement with the physical world, enabling natural and entertaining communication through voice, gestures, and touch, and they are more likely to elicit social behavior from the learners and contribute to their intellectual growth [12]. As a result, social robot-based systems are likely to promote interactive guidance, education, and motivation, which are important facilitators for effective management or prevention of diseases.

Although social robots have been used for several healthcare or educational purposes [13–15], their use toward childhood obesity prevention has been scarce. Rosi et al. [16] applied the humanoid robot Nao in order to improve the nutrition knowledge of children through explanation of concepts and games. In the same direction, Short et al. used a robot in the form of a dragon for nutrition education of children [17]. Barwise et al. [18] utilized a mobile robotic wheel base with an iPad tablet computer, to provide exercise coaching to the children. All the above works do not provide tailored interventions for children according to their particular needs, which may limit the potential of social robots for wide uptake and positive impact on their health behavior outcomes. It is known that tailored health information through a computer interface can result to small but consistent improvements in diet and physical activity behaviors [19], mainly because of its increased relevance to the individual. The main research question of this paper, the answer for which has so far been unknown, is whether tailored recommendations provided by social robot-based systems can trigger changes in the health behaviors of children. Furthermore, an empirical gap remains in testing and validating such interventions in real-world environments.

We present the first-of-its-kind social robot-based platform toward childhood obesity prevention, which uniquely combines: (a) individualized behavioral screening of children, (b) motivation based on varied interactions, and (c) recommendations of goals for health behavior change. The platform is able to collect self-reports of children through the touch of tangible objects and provides personal goal setting in the dimensions of physical activity, diet, and education, in the context of improving children’s health behaviors.

The contribution of this paper is twofold. First, we present the design and development of the social robot-based platform for childhood obesity prevention. In this direction, we provide useful insights into how tailored and interactive platforms with programmable robots can be built in this domain and show the feasibility of screening modifiable behaviors, as well as promoting healthy habits through brief and teachable child–robot encounters. Secondly, we illustrate the evaluation of the developed platform through an experimental study with 30 children in a real-life school setting, demonstrating children’s acceptance, as well as the value and impact of the social robot platform on their health behaviors by following recommended health goals.

## Methodology

### Background and rationale for system design

The design of the system was based on processes of behavior change of the transtheoretical model (TTM) [20], toward providing strategies to guide children which encourage contemplation, preparation, and action for behavior change. These included consciousness-raising to increase self-awareness of children via learning and personal feedback, self-reevaluation to create a positive image of the future self, and reinforcement management to reward and encourage positive behaviors, on the basis of providing brief encounters with children. In this scope, and considering also the findings of previously published research studies with social robots [9, 21, 22], we followed a varied persuasion approach based on natural child–robot interactions, involving the provision of tailored encouragement feedback and individualized goals according to behavioral assessment outcomes (toward consciousness-raising), self-disclosure messages (toward self-reevaluation), as well as motivational hints and entertaining rewards to encourage positive behavior, which could grasp a child’s attention (for example amusing interactions in the form of music and dance).

Overall, the goal was to create an entertaining and motivational system which could engage children in the process of making positive changes on their health behaviors. Specifically, we targeted the behaviors of diet, physical activity, and education because these are considered to be important factors in prevention of childhood obesity [5, 6, 23]. Therefore, we mainly targeted four behaviors according to existing evidence [24]: increasing physical activity, decreasing sedentary activities, reducing unhealthy eating habits, and increasing healthy eating. Goals for those behaviors were targeted on a weekly basis according to goal-setting considerations for persuasive technologies [25]. The educational setting was selected for the operation of the system, because it provides an excellent entry point for reaching children and providing impactful interventions [26]. The age group of children 9–12 years old was chosen, because children at this age are cognitively able to complete questionnaires (for evaluation purposes) and because obesity often starts to rise around this age [27]. The current work builds on initial ideas and preliminary design features of our previous work [28] and extends it through the utilization of a new conceptual framework, new user interfaces for guiding children in their interaction with the robot and the tangible objects, as well as the conduction of an experimental evaluation study with children.

### Conceptual framework

**Fig. 1:**
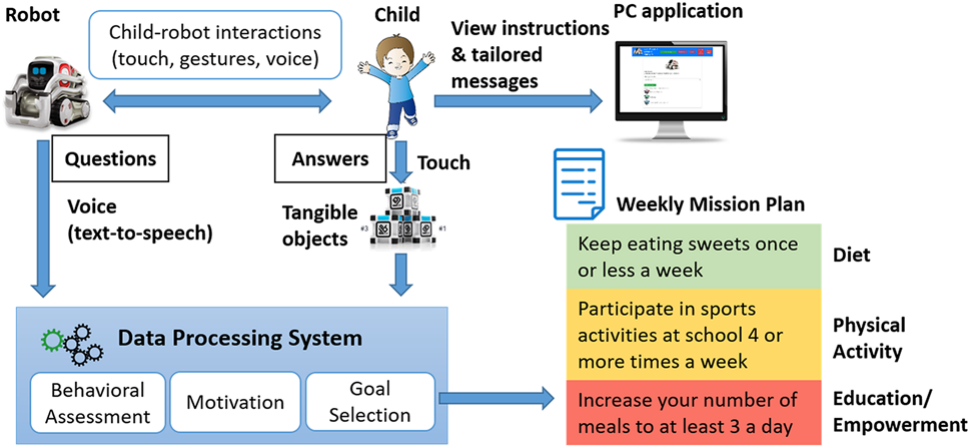
Conceptual framework

The conceptual framework of the social robot-based platform is depicted in Fig. 1. The multi-component platform enables child–robot interactions through using a programmable consumer robot, tangible cubes allowing children to touch them and provide answers to questions asked by the robot, and a PC application to view instructions and tailored messages. A Data Processing System was developed with the following components:

#### Behavioral assessment

This component is responsible for assessing the personal behaviors of the children, and experiential learning. The robot asks the child a series of questions about its health habits. The robot motivates children to change their health behavior through speech (text-to-speech) if this is not optimal or maintain their behavior if this is found to be optimal (motivation component). The questions are provided in the dimensions of diet, physical activity, and education/empowerment, according to reliable questionnaire-based instruments such as the Physical Activity Questionnaire for Children (PAQ-C) [29] and the Food Frequency Questionnaire (FFQ) [30]. The questionnaires are provided as an interactive quiz, in which the answers are given by the child through touching tangible cubes, in order to create a gamified and enjoyable experience [31]. Furthermore, a web application interface (PC application) suitable for display on a monitor was developed, in order to provide written instructions to children, guide them in the process of interacting with the social robot-based platform, and show tailored messages.

#### Motivation

**Fig. 2:**
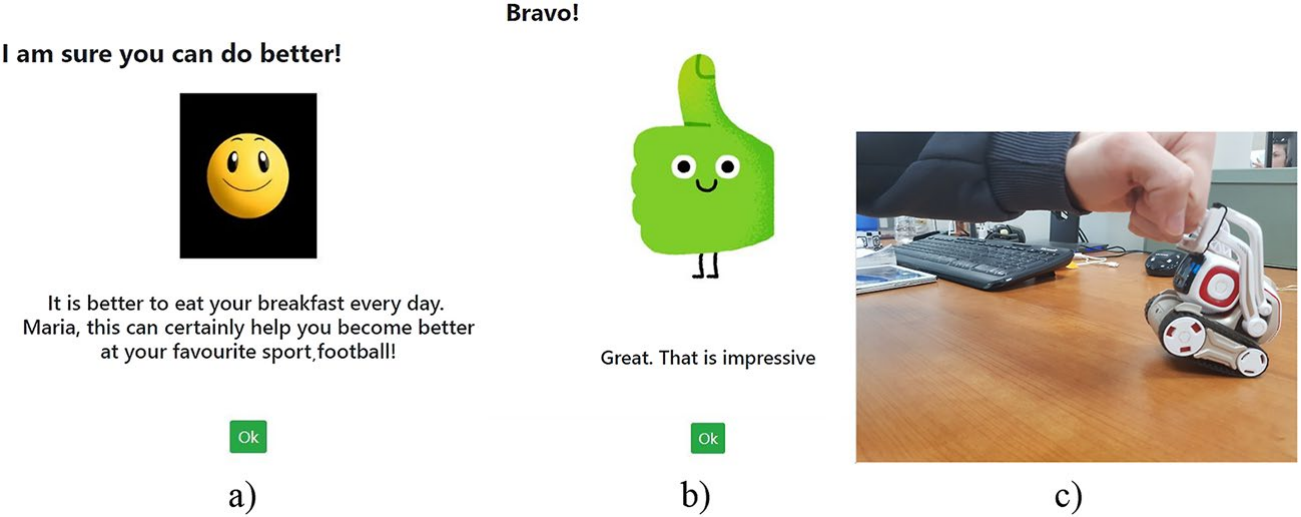
Followed motivational approach: **a** Personalized encouragement feedback, **b** positive reinforcement, **c** natural interaction with the robot to reward behavior—robot raises its arms and asks user to have a fist bump

This component enables the motivation of the child. At an initial step, the social robot asks for the name and favorite sport of the child in order to create a personalized experience, based on which personal features are used for purposes of providing tailored encouragement messages. Then, the system enables sound, kinetic, and facial reactions from the robot, for example, singing, dancing, blinking eyes, fist bump, in order to grasp child attention, further motivate the child, and provide entertaining rewards when the answers in behavioral assessment questions are positive (Fig. 2). Furthermore, the robot responds with speech for education and encouragement purposes (e.g., “well done, fruits are good for your health”, “congratulations for your choice”, etc.), according to our previous work [28]. In this light, the social robot-based platform induces varied feedback and interactions toward making the motivation and education actions less repetitive [32].

#### Goal selection

In this component, following the processing of input behavioral data received within the behavioral assessment component, the child is advised by the robot (through speech) to follow a specific goal (called “mission”) for each of the three dimensions of diet, physical activity, and education/empowerment for the following week. This is achieved according to a red-yellow-green flag (traffic light) decision scheme (according to responses given in the behavioral assessment questionnaires), in which a red flag denotes a behavior which needs immediate change (i.e., prioritized behavior), a yellow flag denotes a not optimal behavior which requires change, and a green flag denoting an optimal health behavior which needs maintenance. An example of mapping of answers in a question to corresponding flags, education/motivation feedback by the robot and missions, is illustrated in Table 1. If two or more behaviors in the same dimension have the same flag, random mission selection occurs at the current implementation stage. However, a more sophisticated logic could be applied for goal selection on the longer-term, e.g., taking into account the analysis of historical data (for example, prioritization of behavior which appeared to need immediate change, i.e., behavior with red flag, most often in the past).

**Table 1:** Mapping of answers regarding breakfast question (diet dimension) to robot feedback and missions

Question	Answer	Education & motivation feedback by robot	Flag	Mission
How many days in a week do you take breakfast?	Everyday	“Great! This is so good for your health.”	Green	“Maintain the number of days having breakfast to 7”
	4–6 times a week	“It is better to eat your breakfast every day. Maria, this can certainly help you become better at your favorite sport, football!”	Yellow	“Increase the number of days having breakfast to 7. You can do it!”
	3 or less times a week	“Maria, it is better to eat your breakfast every day.”	Red	“Increase the number of days having breakfast to 4 or more. You can do it!”

### Technical infrastructure

**Fig. 3:**
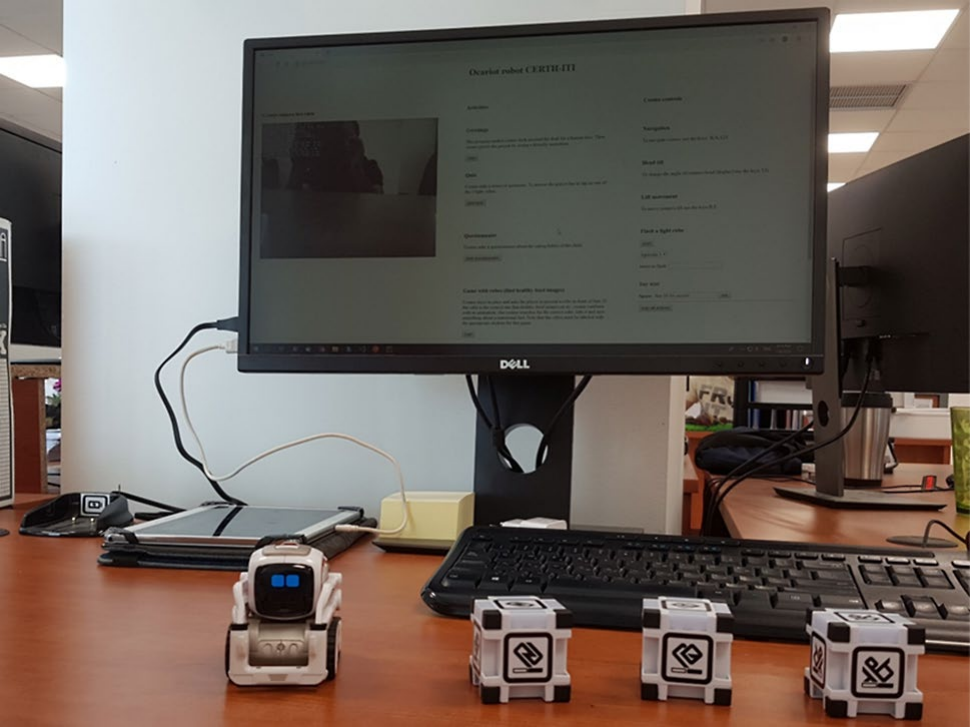
Social robot platform components

**Fig. 4:**
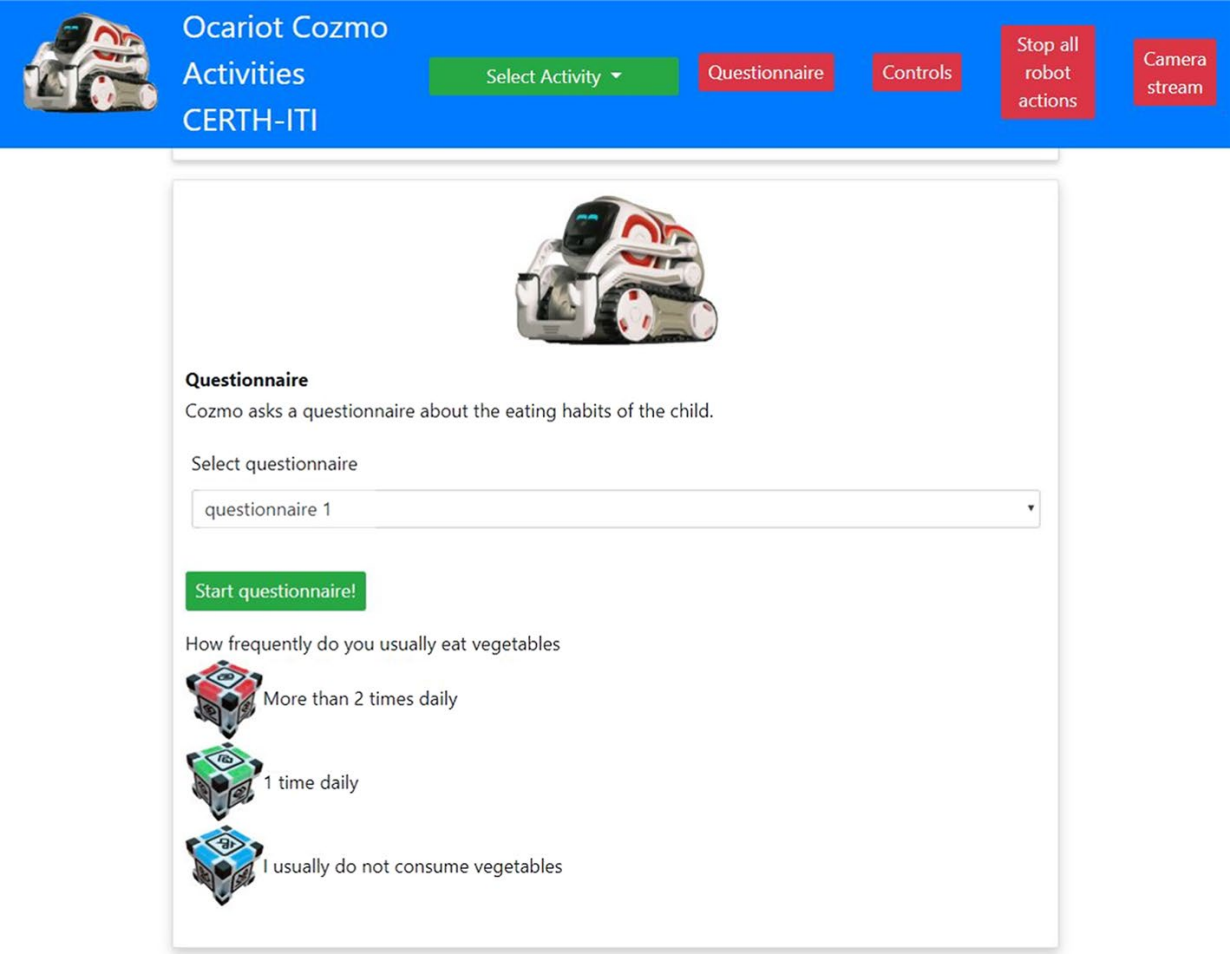
PC interface to guide children in their interaction with robot and tangible objects

The social robot platform and the flow of child–robot interactions are demonstrated through a published YouTube video.^1^ We used the Cozmo robot in the development of our system^2^ (Fig. 3). We used Cozmo mainly because it is a programmable social robot via a Standard Development Kit (SDK) in Python, facilitating rapid prototyping and support through several libraries. Furthermore, Cozmo has useful features such as capability to speak via text-to-speech instructions, and programmable eye animations to show feelings which were useful in the creation of multimodal interactions [33]. In addition, Cozmo is able to interact with three small tangible cubes which are part of the platform and empowered with accelerometers, which have been programmed in this work to act as a medium of children’s answers to the robot’s questions. In this light, upon touching one of the three cubes, according to the instructions provided by the robot, a corresponding answer to a question is forwarded to the Data Processing System. The SDK enabled us to program asynchronous events required, e.g., in the interaction with its cubes (i.e., when a child touches them). The PC application user interface allowed children to view the instructions and questions provided by the robot in written form during the interactions (used for example in case they did not understand/heard well a question), thereby contributing to a more accessible and easy-to-use system. It was developed with the scalable Angular web application framework (Fig. 4).

The questions asked by the robot were formed to acquire personal behavioral data, e.g., how many fruits do you usually eat? The list of questions asked by the robot for behavioral assessment can be found in “Appendix 1”. Based on our traffic light decision scheme and according to the answers given in the questions, three in total weekly goals (“missions”) in the dimensions of diet, physical activity, and education/empowerment, requiring the most attention, are announced by the robot, and displayed to the PC application. For example, a mission in the diet dimension would be “Try to eat at least 3 fruits/day”, for a child answering 1 or 2 fruits daily (“yellow flag”) in the corresponding behavioral question (Q2 in “Appendix 1”). The missions proposed by the robot are also printable, as a “take home” message for the children.

**Fig. 5:**
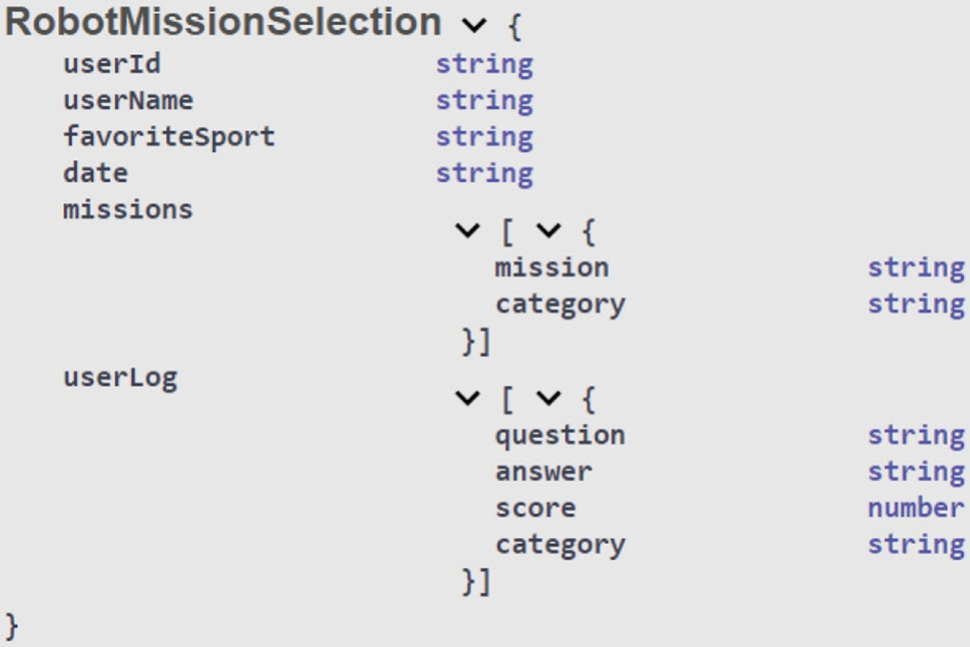
Data model of mission selection in JSON based on children’s answers

In order to promote the interoperability and extensibility of our platform, we adopted a REST (Representational State Transfer) microservice architecture, enabling to integrate its operations in other systems. The microservice architecture is a recent architectural style for web services that structures an application into a set of small, independently deployable microservices, offering flexibility and scalability [34]. The mission selection by the social robot is supported through two REST API calls, one for posting the missions selected, and another for getting the last missions. The generic data model in JSON (JavaScript Object Notation) format can be seen in Fig. 5. The model is simple and also supports tracking changes over time (through the “date” attribute). The mission selection APIs have been integrated within the OCARIoT (Obesity Caring Solution using IoT potential) platform, a multi-component platform based on wearable devices, a mobile app for children, and a dashboard for carers, toward preventing childhood obesity [35].

## Results

### Experimental evaluation study

In this section, we present the experimental evaluation study to assess whether the developed social robot-based platform is acceptable by the children and has a positive impact on their adoption of healthy behaviors. The study was conducted in a public school in Thessaloniki, Greece, under the supervision of teachers and researchers. Consent forms were signed by parents prior to children’s participation, providing detailed information about the aims and procedures of the study. Children at the age of 9–12 years with no mental health disease, with the written approval of their parents, were randomly selected by teachers for their participation using a random number generator. At any stage of the study, the child could leave the study. The study was conducted in Greek.

Each child participating in the study was expected to have a 20-min interaction with the social robot platform. In the beginning, the researcher asked the child whether he/she has ever seen or used the Cozmo robot before, and then completed the first name and favorite sport of the child in the platform. Then, the robot asked the child questions related to diet, physical activity and education/empowerment toward its behavioral assessment (see list of questions in “Appendix 1”), and according to the answers, it provided encouragement, education, and interactive entertaining rewards (such as a ballet dance or a Christmas song performed by the robot) according to our motivation approach. The final step in the flow of child–robot interactions was the provision of three missions (one in each dimension), which were printed and asked by the robot to try to achieve for the following week.

After the end of the child–robot interactions, children completed a brief questionnaire in a faces scale (1–5 scale) to assess the acceptability of the platform in a single encounter. A faces scale was used because it is preferred by children and it is considered to be appropriate [36].

In accordance with acceptance models for social robots [37], we wanted to assess the attitude of participants (Q1: Was the robot interesting to you?) and investigate whether children perceived the robot as able to understand (Q2: Did the robot show that it can understand?), because previous results have shown that participants appreciate a socially intelligent agent more and have a higher intention of using it [38]. Furthermore, we assessed the perceived enjoyment of the children (Q3: Did the robot make you happy or smile/laugh?), which is associated with making a technological product compelling [39]. An item for perceived usefulness of the robot in the context of healthcare was also included (Q4: Can the robot help you to have better health?).

After seven days, a follow-up visit at the school took place to provide a printed questionnaire with the 14 questions for behavioral assessment (“Appendix 1”) to the participating pupils. The goal was to explore whether the health behavior of children improved within a week in relation to the missions they received through the social robot platform. In order to reduce bias during the follow-up assessment, all 14 questions of “Appendix 1” were asked, and not only those mapping to the 3 specific missions of each child. Summary statistics for goal achievement were used, and the two-proportion z-test was utilized to identify possible gender differences.

### Study outcomes

In total, 34 children participated in our study from 4 grades, i.e., 3rd grade (10 children), 4th grade (7 children), 5th grade (8 children), and 6th grade (9 children). In total, 10 classrooms were involved. One child dropped out because of family reasons, and 3 children were absent during the follow-up visit at the school. Therefore, herein the outcomes from 30 children are presented.

**Fig. 6:**
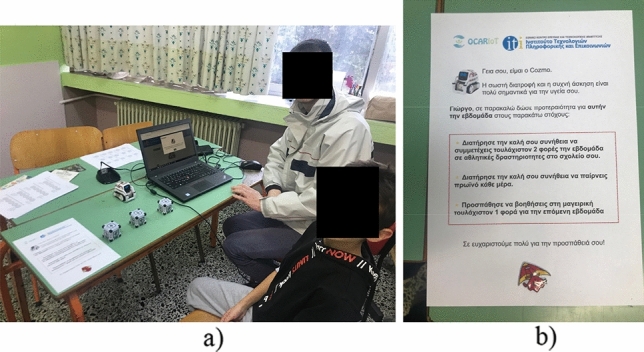
**a** Child interacting with the social robot at the school setting, **b** printed mission sheet for health behavior change as a “take home” message

Of the 30 children, 17 were boys and 13 were girls. The mean age was 10.5 ± 1.2 years. None reported to have ever used or seen the Cozmo robot before. The mean scores in the acceptability questionnaire were 4.9 ± 0.1 for Q1, 4.8 ± 0.4 for Q2, 4.8 ± 0.3 for Q3, and 4.6 ± 0.5 for Q4, thereby showing that the robot was very interesting for the children, capable to understand, able to make them happy, and helpful in achieving better health (Fig. 6).

The missions recommended most for children were on trying to spend less than 1 h in front of a screen with 10 occurrences (33%) in the physical activity dimension, increasing fish consumption to 2 or more times a week with 5 occurrences (16%) in the diet dimension, and trying to participate in meal’s preparation at least 1 time a week with 8 occurrences (26%) in education/empowerment dimension. The occurrence of most recommended missions along with their achievement rate according to the follow-up behavioral assessment can be found in Table 2.

**Table 2:** Missions recommended most for children and their achievement rate

	Mission	Flag	Occurrences in 30 children (% percentage)	Achievement Rate (%)
Physical activity	Try to spend less than 1 h in front of a screen	Yellow	10 (33%)	9/10 (90%)
	Keep participating in sports activities at school for 2 or more times a week	Green	8 (26%)	8/8 (100%)
	Try to participate in sports activities outside school for at least 2 times a week	Yellow	5 (16%)	4/5 (80%)
Diet	Increase fish consumption to 2 or more times a week	Yellow	5 (16%)	4/5 (80%)
	Increase fruit consumption to 3 times or more daily	Yellow	4 (13%)	4/4 (100%)
	Maintain the number of days having breakfast to 7	Green	4 (13%)	4/4 (100%)
Education/empowerment	Try to participate in your meal's preparation at least 1 time a week	Yellow	8 (26%)	6/8 (75%)
	Increase the number of meals per day to at least 4	Yellow	5 (16%)	4/5 (80%)
	Try to go to the supermarket with your parents at least once a week	Yellow	3 (10%)	3/3 (100%)

**Fig. 7:**
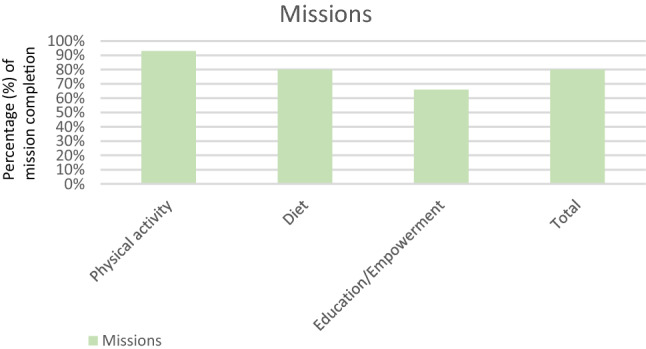
Percentage of mission completion by children after 1 week

The follow-up behavioral assessment revealed that 80% (72/90) of the recommended missions to the 30 children were achieved (Fig. 7). More specifically, 93% (28/30) of physical activity missions, 80% (24/30) of diet missions, and 66% (20/30) of education/empowerment missions were achieved. The number of achieved missions requesting improvement on health behavior and not maintenance was almost half, 35 out of 72 (48%). Some 77% (23/30) of the children had at least 1 mission requesting improvement of behavior, accomplished. As seen also in Table 2, missions which had several occurrences in children and requested improvement of behavior, such as missions related to reducing the time spent on a screen (90% achievement rate), increasing fish consumption (80% achievement rate), and participating in meal’s preparation (75% achievement rate), had a high achievement rate. Interestingly, 89% (35/39) of the missions for girls were accomplished, compared to 72% (37/51) of the missions for the boys, denoting a statistically significant difference (*p* = 0.02).

## Discussion

A social robot-based platform toward health behavior change and prevention of childhood obesity was presented. The primary finding of this paper is that a multi-component system based on a social robot for assessing health behaviors, motivating children and guiding them toward achieving personal behavioral goals, is technically feasible, acceptable by children, and able to facilitate betterment of children’s health behaviors. To our knowledge, there has not been a similar social robot-based system, which has been developed and evaluated in a real-life setting.

The social robot platform development showed that such multi-component systems in the area of childhood obesity prevention can be feasible through using commercially available robots, tangible items for the interaction of the children, and web applications for augmenting the child’s experience. Such a platform setup provides means of varied interaction and is able to aid children’s understanding in robot–child interactions, motivate them, and promote their satisfaction. In this direction, we took advantage of the programmable robot’s in-built capabilities such as text-to-speech synthesis and movement, to provide a stimulating platform which requires minimal child interactions.

The deployment of our platform has revealed that social robot-based systems can be effectively utilized to collect children’s self-reports for health purposes, which is first and foremost required toward their behavioral screening. Social robots with talking, guiding, and rewarding functions such as the one developed in this study are engaging, which can motivate children to become more interactive and collaborative in this regard. Nevertheless, it is evident that further longitudinal studies are needed to show the level of engagement of children with such a system [32]. Those future studies could also reveal the most effective persuasion means toward sustained health behavior change.

The experimental evaluation study with 30 children illustrated that the social robot-based platform was highly interesting to the children, entertaining, and helpful in their health promotion. The platform proved to be a useful starting point toward health behavior change, since 80% of the missions proposed by the robot were accomplished by the children in a week, and 77% of the children had at least one achieved mission requesting improvement of a health behavior. Girls were found to achieve missions in a greater degree than boys, possibly because of their higher interest in strategies to lose weight [40].

The study outcomes provide preliminary evidence of the usefulness of social robots in childhood obesity prevention. Furthermore, they demonstrate the need to conduct further longer-term rigorous studies with social robot-based interventions (with a large effect size), considering the challenge of sustaining health behavior change over a long period of time on the one hand [41], and the scarcity of such studies in child healthcare on the other [42]. The recent COVID-19 outbreak, which forced children around the globe to be isolated at home and change their health habits due to quarantine policies, further makes the use of such novel interventions more timely and necessary.

Our future work involves the conduct of studies with children to show the degree of their engagement with social robots on the long term. In this direction, harnessing sufficient historical behavioral data could possibly reveal patterns on health behavior change and make personalized coaching interventions based on a decision support system possible [43]. Furthermore, we aim to enhance child–robot interactions through incorporating speech recognition, thereby enabling child–robot dialogues which could be used for persuasion purposes [44]. Automated diet tracking by leveraging the robot’s camera to recognize food will also be integrated in line with our previous work [45]. Finally, future work involves the deployment of the platform at different settings, such as hospitals and pediatric centers [46], to collect children’s health self-reports and guide them toward the effective management of obesity or other conditions requiring lifestyle changes.

### Limitations

The outcomes of the study described herein should be interpreted in the context of its limitations. First, the interaction of the social robot platform with the children was brief within a one-time encounter, and we examined effects on health behaviors only in a 7-day period. Multiple interaction encounters and a longer follow-up would be needed to provide more robust evidence on whether children could maintain or improve their health behaviors as time progresses. However, based on the outcomes of our study and considering the usefulness and acceptance of previously developed digital screeners [46], we believe that the use of such a social robot-based intervention could be a useful starting point for triggering behavior changes in children. Furthermore, recall bias is a common issue with questionnaire-based tools as employed in this study, and no mitigation measures were taken in this regard. Recalling health behaviors such as physical activity during the past 7 days is a complex cognitive task for children, which might have led to their overestimation or underestimation [47]. No control group was included to assess the impact of the social robot-based intervention. The sample size was small (30 children), which prevented to evaluate the effectiveness of single components alone and test between conditions in a more refined way (i.e., by capturing smaller differences in terms of effect size). The small sample size also prevented the identification of possible statistically significant differences among children of different age. Furthermore, this study was conducted in a supervised environment (school). It is uncertain whether children could use such a system on their own without any help, e.g., at their home. Parents were not surveyed in this study. However, their role in promoting children’s healthy behaviors could be significant [48], requiring investigation in future work.

## Conclusion

We presented a novel social robot-based platform toward childhood obesity prevention. The platform was found to be interesting and useful by the children, through testing in a real-life school setting. The present work provides preliminary evidence on the feasibility and usefulness of social robot-empowered interventions to screen and guide children for health behavior change within brief child–robot interaction encounters. Further rigorous studies are now required to provide more robust evidence on the impact of social robots on children’s health behaviors.

## Data Availability

The datasets generated during and/or analyzed during the current study are available from the corresponding author on reasonable request.

## Footnotes

1 Social robot platform demonstration: https://www.youtube.com/watch?v=DT8W3iwBLcw.

2 Cozmo robot: https://anki.com/en-us/cozmo.html.
